# Leveraging genomes to support conservation and bioeconomy policies in a megadiverse country

**DOI:** 10.1016/j.xgen.2024.100678

**Published:** 2024-10-17

**Authors:** Sibelle Torres Vilaça, Amanda F. Vidal, Ana Carolina D’Oliveira Pavan, Bruno Marques Silva, Carolina S. Carvalho, Cintia Povill, Danielle Luna-Lucena, Gisele L. Nunes, Henrique Vieira Figueiró, Izabela Santos Mendes, Jose Augusto P. Bittencourt, Lara Gomes Côrtes, Lucas Eduardo Costa Canesin, Renato R.M. Oliveira, Roberta P. Damasceno, Santelmo Vasconcelos, Silvia B. Barreto, Valeria Tavares, Guilherme Oliveira, Amely Branquinho Martins, Alexandre Aleixo

**Affiliations:** 1 Instituto Tecnológico Vale, Belém, PA 66055-200, Brazil; 2 Centro Nacional de Pesquisa e Conservação de Répteis e Anfíbios, Instituto Chico Mendes de Conservação da Biodiversidade, Goiânia, GO 74605-090, Brazil; 3 Centro Nacional de Pesquisa e Conservação de Primatas Brasileiros, Instituto Chico Mendes de Conservação da Biodiversidade, Cabedelo, PB 58108-012, Brazil

**Keywords:** biodiversity, monitoring, capacity development, decolonization, essential biodiversity variables, Global South, Kunming-Montreal Global Biodiversity Framework

## Abstract

The biodiversity crisis is a global phenomenon, and measures to monitor, stop, and revert the impacts on species’ extinction risk are urgently needed. Megadiverse countries, especially in the Global South, are responsible for managing and protecting Earth’s biodiversity. Various initiatives have started to sequence reference-level genomes or perform large-scale species detection and monitoring through environmental DNA. Here, we outline the Genomics of the Brazilian Biodiversity (GBB) consortium that is contributing to public policies on the conservation and management of Brazilian species. We describe our unique public-private governance and lessons in setting up a genomic consortium in a megadiverse country of continental scale. We explore the challenges while sharing knowledge for similar initiatives in the Global South. Ultimately, we aim to encourage Brazilian institutions and other megadiverse countries to invest and participate in large-scale genomic initiatives, demonstrating their commitment to preserving and monitoring their exceptional natural heritage while contributing to global biodiversity conservation.

## Graphical abstract

**Figure:**
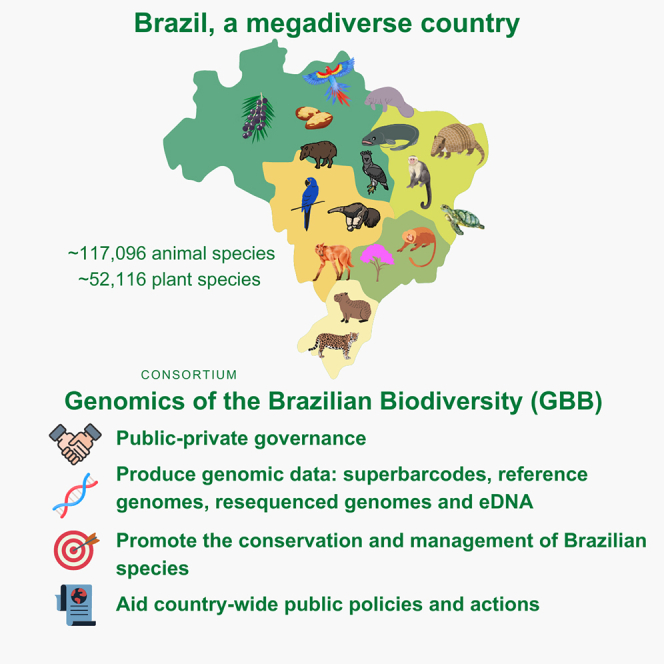

## Teaser

Vilaça et al. described a unique private-public initiative, the Genomics of the Brazilian Biodiversity (GBB) consortium. They outline the goals and challenges faced so far and discuss the gaps concerning the generation, analyses, usage, and capacity building in genomics between countries in the Global North and South.

## Introduction

Megadiverse countries, especially in the Global South, safeguard and protect an astonishing extent of the planet’s biodiversity. Brazil, the fifth largest country, hosts 15%–20% of the world’s biological diversity, including 117,096 known animal species.^1^ Among vertebrate species, Brazil has the highest number of amphibians and primates in the world, second in mammals, and third in non-avian reptiles and birds.^1^ From a total of 52,116 plant species estimated to occur in the Brazilian territory,^2^ an extraordinary number of over 17,000 are endemic.^3^ Unfortunately, the rate of biodiversity loss due to anthropogenic activities such as habitat fragmentation and degradation has been disproportionate compared to actions toward its protection, compounded by an acceleration in habitat degradation over the last few years.^4^^,^^5^ Developing immediate and effective conservation policies to preserve biodiversity is crucial not only for Brazil’s ecological stability but also for global conservation efforts.

Biodiversity monitoring using genetic diversity parameters has gained new breath with the signing of the Kunming-Montreal Global Biodiversity Framework (KMGBF) during the United Nations Biodiversity Conference (15^th^ meeting of the Conference of the Parties to the Convention on Biological Diversity [COP15]) in December 2022.^6^^,^^7^ Indicators of long-term genetic and evolutionary resilience are one of the long-term goals of the KMGBF (Goal A), with action-oriented global targets for urgent action until 2030 (Target 4) and associated indicators (e.g., headline indicator A.4 “The proportion of populations within species with an effective population size > 500”) that benefit from genetic data.^8^^,^^9^^,^^10^^,^^11^ However, DNA-based data for endangered species such as barcode references (i.e., mitogenomes and plastomes), chromosome-level reference genomes, and assessments of long-term genetic health using genomic methods are lacking, especially in megadiverse countries. This scenario has stimulated Brazilian public and private institutions to invest in a large-scale genomic initiative, demonstrating their commitment to preserving and monitoring the exceptional natural heritage found in the country while contributing to global biodiversity conservation.

**Figure 1:**
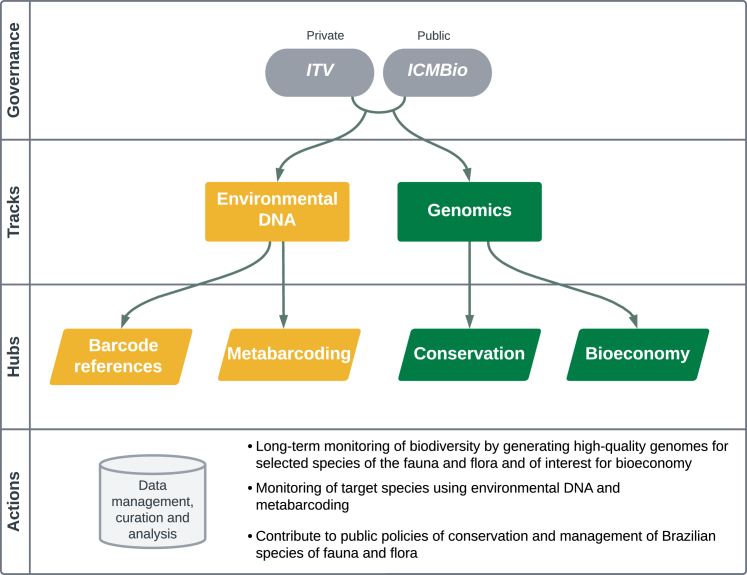
Schematic diagram of the governance and research tracks of the GBB The consortium’s governance is a private-public partnership between a non-profit research institute (Instituto Tecnológico Vale [ITV]) and the Instituto Chico Mendes de Conservação da Biodiversidade (ICMBio). The two main research tracks are divided into two hubs with unique goals and methodologies.

In the past decade, various global initiatives have started sequencing reference-level genomes or performing large-scale monitoring through environmental DNA (eDNA). In January 2023, a nationwide initiative was launched in Brazil to generate high-quality and high-quantity genomic data to support conservation actions and unlock its bioeconomy potential. Our unique private-public partnership, entitled Genomics of the Brazilian Biodiversity, hereafter GBB (“Genômica da Biodiversidade Brasileira” in Portuguese; https://www.itv.org/en/genomics-of-the-brazilian-biodiversity-gbb/), has set ambitious goals toward data production and capacity development (Figure 1). An essential aspect of food security is conserving biodiversity; thus, sustainable development depends on preserving the flora and germplasm of economically important species.^12^ Unlocking the value of native species inclusively and sustainably while recognizing and valuing traditional knowledge guarantees equality in economic opportunities and is a matter of national strategic significance for the country’s development. Here, we share experiences that provide an outlook of the future for other Global South and megadiverse countries in the pursuit of setting up biodiversity-focused genome networks.

## GBB overview and governance

The GBB is a Brazilian consortium aimed to create a collaborative network for applied research in biodiversity conservation and support bioeconomy development based on native species. It aims to attend to joint needs for nationwide biodiversity-related policies for which DNA data are needed. This nationwide network will leverage genomic data on species of conservation and economic interest and establish genomic protocols for biodiversity monitoring. In its conception, the main actions of the consortium are as follows.

(1) Structuring a national network for generating barcode references (i.e., mitogenomes and plastomes) for species identification.
(2) Sequencing high-quality genomes for selected species of fauna and flora, including those of interest for the Brazilian bioeconomy.
(3) Resequencing genomes for (1) estimates of population structure and genetic diversity for wildlife and captive species of conservation concern and (2) associations of phenotypic and environmental traits of species of interest for the bioeconomy.
(4) Monitoring target species/biological communities using eDNA.

Specifically, the GBB is divided into two research tracks: genomics and eDNA, each further divided into two hubs (Figure 1). The GBB governance is a private-public partnership between a non-profit research institute (Instituto Tecnológico Vale [ITV]) and the Instituto Chico Mendes de Conservação da Biodiversidade (ICMBio). While ITV is funded by Vale, a Brazilian multinational mining company and one of the largest logistics operators in the country, ICMBio is a branch of the Brazilian Ministry of Environment and Climate Change (MMA) responsible for proposing, implementing, and managing conservation policies regarding the Brazilian federal protected areas (PAs), sustainable use of natural resources, threatened species, and research for biodiversity conservation.

In 2023, the GBB consolidated its internal governance policy, whereby the decision concerning the projects executed under the consortium is primarily the responsibility of the governmental partner (ICMBio) and based, for the most part, on already-existing national action plans (NAPs; or “PANs” in Portuguese) for the conservation of threatened species (see below), which in turn were consolidated previously and are reviewed periodically by hundreds of ICMBio staff and Brazilian personnel from academic institutions, non-governmental organizations (NGOs), and civil society. ITV is primarily in charge of supporting the design and execution of these projects from a scientific perspective, along with the participation of, thus far, dozens of NGOs and academic collaborators from throughout the country.

## Choosing targets for genomics projects in Brazil

Research is crucial for conservation policies, and ICMBio’s attributions and monitoring goals depend on robust data, including genomics. To define priorities for GBB’s data production, members of the ICMBio who integrate the GBB’s governance engaged in internal consultations involving representatives of all the 14 ICMBio research centers and 334 PAs. This collaborative approach, which also involved the scientific community, was designed to assess and produce genomic data for species of significant national importance, focusing on the broader goal of biodiversity conservation and socio-economic inclusion of traditional communities. As a result, over 400 projects related to reference genomes, population genomics, and eDNA monitoring were listed.^13^ In our exercise to prioritize species and areas for genome sequencing and eDNA monitoring, the necessity and importance of DNA-based data permeating conservation needs within Brazil became clear. Notably, these demands target specific conservation needs from government staff with technical expertise in all Brazilian biodiversity, ranging from single species to entire biomes. This crucial country-wide survey of scientific questions and monitoring gaps that require genomic data reflects the necessity of basic knowledge from Brazil’s biodiversity and also the establishment of vital large-scale DNA-based monitoring in this megadiverse country.

After a 4-day-long group discussion held in September 2023, the conservation genomics hub decided to prioritize projects focusing on (1) imperiled species evaluated within the System of Extinction Risk Assessment (SALVE) database system^1^^,^^14^ based on their extinction risk according to the IUCN Red List categories and criteria (https://www.iucnredlist.org/resources/categories-and-criteria); (2) species with no available genetic data; (3) species in need of genomic data to support management, as listed by the ICMBio Research Centers, a strategy that will maximize overall taxonomic coverage; and (4) endemic species. Concerning bioeconomy, many native Brazilian species widely consumed, like cocoa (*Theobroma cacao*) and açaí (*Euterpe oleracea*), are commercially exploited by local Indigenous Peoples and traditional communities. To facilitate the management of understudied crops and contribute to generating data on complex traits, we will employ a multi-omics approach for genotype-phenotype association targeting traits of interest. Additional native species exploited commercially by local communities in PAs under the ICMBio governance (e.g., the fish arapaima or pirarucu, *Arapaima gigas*) will be added as targets from 2024 onwards.

## Challenges of conducting genomics in the Global South

The GBB is an unfolding of a previous genome project, AmaZOOmics, a 2-year collaboration between ITV and the Vertebrates Genome Project (VGP) that started in 2021 and aimed to assemble 29 high-quality genomes from Amazonian vertebrates. However, exporting high-quality samples to the VGP hub in the USA took 1 year and 8 months due to the time needed to obtain permits for sampling and the international transit of biological material of CITES-listed species between Brazil and the USA. Shipping samples to sequencing centers overseas has proved to be no less challenging and costly, as few transport companies guarantee the required cold chain in dry ice within the country and abroad, a requirement for sequencing and assembling chromosome-level genomes.

Given the challenges in exporting samples for sequencing high-quality genomes, the next natural step was to sequence genomes in Brazil. The ITV lab in Belém serves as the core lab for the GBB and currently has a PacBio Sequel IIe, a Nanopore PromethION P2, multiple Illumina sequencers, and a state-of-the-art molecular biology lab. As of today, in Brazil, only four PacBio IIe sequencers are running. The amount of machines in-country and, in fact, in Latin America (only one other PacBio long-read sequencer is operating in Mexico) by itself makes it challenging to obtain training and maintenance of the equipment, as very few people can operate and service it. Moreover, other sequencing companies do not provide equipment maintenance services through their Brazilian sales representatives.

Like most emergent and developing nations, Brazil experiences high operating costs for scientific laboratories because most reagents and equipment are imported from the Global North.^15^ The cost of sequencing a 2 Gb chromosome-level genome with HiFi reads in Brazil is 3.3× higher than in the USA (Table 1). Furthermore, the delivery of consumables often encounters challenges, with kits that require refrigeration frequently arriving at room temperature due to delays in delivery or customs clearance.

**Table 1:** The cost of doing genomics in Brazil

	Brazil (USD)	USA (USD)	BR/USA ratio
PacBio reads (60×)	17,401.20	5,223.00	3.33
Hi-C (Arima) kit for 8 reactions	4,395.60	3,062.00	1.43
Illumina reads (60×)^a^	5,258.30	2,050.00	2.56
Nanopore reads (60×)	4,075.66	1,350.23	3.02
PacBio Revio	1,281,688.13	779,000.00	1.64
Nanopore P2 Solo Basic	51,000.00	17,100.00	3.00
Storage (1 Petabyte)	93,622.09	80,960.00	1.16

Prices practiced in Brazil in Brazilian Reais (BRL) were converted to US dollars (USD; conversion rate BRL 1 = USD 0.2) for comparison. Prices are as of July 2023. We considered a 2 Gb genome for coverage calculation. BR, Brazil.

a Illumina NextSeq 1000/2000.

The current standard for reference genomes from the EBP and VGP^16^^,^^17^ uses a combination of long-read technologies, like PacBio HiFi, and chromatin conformation capture data (Hi-C). Despite being mentioned in EBP’s standards,^17^ other technologies, like Oxford Nanopore, have not been used as often to produce high-quality genomes.^18^ Obtaining kits and reagents for Illumina and PacBio sequencing is feasible and timely; however, this is not the case yet with Hi-C kits, which are fundamental for producing chromosome-level genome assemblies. No companies that sell widely implemented Hi-C kits have commercial representatives in Brazil, requiring direct import from their USA office.

While GBB is privately funded by Vale for ∼$25 million until 2027, we recognize that a project of this magnitude would likely not happen yet if exclusively dependent upon government funding. One of the largest competitive funding programs from the Brazilian Council for Scientific and Technological Development (CNPq) aims to fund strategic networks that tackle broad national issues through high-impact science and technology research (i.e., the National Institutes of Science and Technology [INCT]). The 2023 funding call for the INCT program had a cap funding of $1,400,000 for 5-year projects, an amount hardly sufficient for acquiring a PacBio Revio sequencing machine (Table 1). Nevertheless, there are grant calls in Brazil for the acquisition of large equipment, while there are very few calls that grant funding for their maintenance (e.g., Brazilian Innovation Agency [FINEP] calls). It is not uncommon for Brazilian institutions to have expensive equipment acquired through grants not being used due to the lack of subsidy for maintenance or spare parts.

Finally, private-public enterprises like the GBB are proven alternatives for tackling the inherent high costs of large-scale genome projects throughout the world, particularly in Global South countries. Transnational corporations, in particular, can join efforts with governmental, education, environmental, and scientific institutions at the local and national levels to leverage biodiversity genomics initiatives. These joint efforts are set to leave a long-lasting positive legacy on the economy, environment, and capacity building.^19^

## Equitable actions

**Figure 2:**
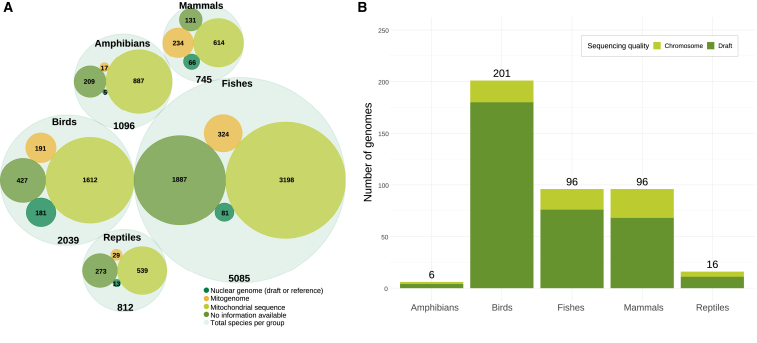
Representativeness of genetic and genomic data for Brazilian vertebrate species (A) Representativeness of genetic data for Brazilian vertebrate species. Each group has a total number of species (outer circle) divided by the different types of genetic or genomic data available (inner circles). Mitochondrial sequences refer to any segment of mitochondrial data available on GenBank, while mitogenomes are separated into their own category. (B) Representativeness of chromosome-level and draft nuclear genomes for vertebrates occurring in Brazil. If a single species had draft and chromosome-level genomes, both were included in our dataset. The species list was retrieved as per the System of Extinction Risk Assessment (“Sistema de Avaliação do Risco de Extinção da Biodiversidade”), hereafter SALVE, a database used to support the assessment of Brazilian fauna conservation status and organize information about the assessed species. SALVE’s list of assessed species for extinction risk considered a total of 9,777 vertebrates. The data used were downloaded from the NCBI GenBank public database on August 29^th^, 2023.

**Figure 3:**
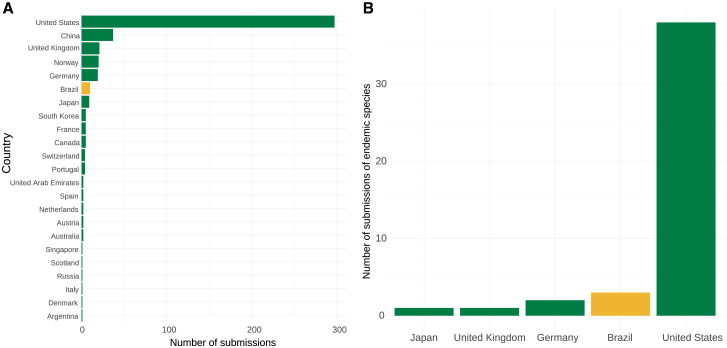
Overview of the countries that have submitted to NCBI genomes of Brazilian species (A) Countries that deposited reference genome sequences from vertebrate species occurring in Brazil and evaluated by ICMBio through the SALVE assessment. (B) Number of reference genomes sequenced from species endemic to Brazil submitted to the NCBI database, categorized by the country of the submitting organization. Data for both graphs were obtained according to the submitter’s country. In cases where the submitter’s country was unclear, the associated BioProject description was used. The species list was retrieved from the SALVE database considering a total of 9,777 vertebrates. The data used were downloaded on August 29^th^, 2023, from the NCBI GenBank public database.

Brazil has had a national access and benefit-sharing framework since 2001, with a national system on genetic resource management and associated knowledge implemented (SISGEN) since 2015 (law 13.123/2015). More recently, Brazil was the 130^th^ country to ratify the Nagoya Protocol in 2021 (legislative decree no. 136/2020 published in the Federal Official Gazette on August 12^th^, 2020).^20^ Despite these advances, Brazil and other Global South countries need to engage in national and multinational genomics consortia not merely as providers of raw biological material intended for sequencing but as contributors of skilled human resources that should be actively trained under these initiatives. However, given the discussed struggles in producing genomic data for Brazilian species (Figure 2), a large portion of studies associated with genomics have their data produced outside the country (Figure 3). Besides fostering interdisciplinary collaborations, knowledge exchange, data sharing, and technological advancements in genomic methods, the GBB will promote capacity building and disseminate knowledge of genomics to ICMBio staff and the wider community of Brazilian researchers.

Another relevant equitable action will be the active participation of Indigenous Peoples and local communities (IPLCs) in genomic studies focused on species of bioeconomic interest through the integration between GBB and the Sustenta Bio initiative, carried out by the Brazilian Ministry of the Environment and Climate Change (MMA), Vale, and the Vale Fund. In short, Sustenta Bio aims to strengthen productive chains linked to species of bioeconomic value in 15 territories of local communities covering ∼10 million hectares in the Brazilian Amazon. GBB will act as a genomics solution hub supporting Sustenta Bio initiatives, initially focusing on the açaí, pirarucu, and Brazil nut (*Bertholletia excelsa*) chains. This joint entrepreneurship predicts the implementation of benefit-sharing mechanisms by local communities related to the innovation of productive chains involving bioeconomy products in their territories and beyond. Within this initiative, we also aim to address KMGBF’s target 9 on the sustainable use of wild species by IPLCs and target 13 on the use of genetic resources and digital sequence information (DSI) for traditional knowledge and benefit sharing, including capacity building.

Finally, all generated data will be shared according to FAIR (findable, accessible, interoperable, and reusable) and CARE (collective benefits, authority to control, responsibility, ethics) principles. All sampling and scientific knowledge generated will abide by the national and international laws on genetic heritage and benefit sharing discussed above. Given GBB’s shared governance, a key goal is capacity building in genomic methods of the ICMBio staff responsible for country-wide conservation policies. This will not only facilitate our goals to sequence and analyze thousands of genomes and translate the results to conservation actions but also guarantee the appropriation of government officials directly involved in those actions to delineate studies, gather samples, and analyze data.

## Decolonizing biodiversity genomics

As we have embarked on this ambitious journey, we recognize the many challenges ahead for generating a genetic/genomic catalog of Brazilian biodiversity that is also shared with other megadiverse countries in the Global South. By leveraging advanced genomic technologies, the GBB is committed to being a launchpad for a deeper understanding of Brazil’s unique ecosystems, facilitating policy decision-making, and establishing a foundation for sustainable management practices while guaranteeing the country’s sovereignty by obtaining, processing, and analyzing its own data. Scientific decolonization is done in steps and needs to embrace local knowledge, access to infrastructure, and expertise.^21^ Research funds for large-scale genome sequencing have largely been geopolitically bound and have not included priority areas outside the origin of the funding source.^19^^,^^22^^,^^23^ At best, training was offered,^24^ but capacity building alone is not sufficient to establish a long-lasting local infrastructure employing continuously skilled personnel working routinely with genomic datasets. Research agendas, especially involving innovation and technology (like genomics), are often politically bound.^23^^,^^25^ Even if funding for local sequencing capacity is intended to be built, our challenges in importing and acquiring reagents exemplify that sequencing genomes outside of the Global North is not a trivial effort. As a consequence, genomes for Brazilian species are mostly sequenced abroad (Figure 3). In cutting-edge fields, research and development (R&D) investments are an important spillover catalyst for encouraging innovative areas, from human capital to biotechnology, and Brazil has low investments in R&D compared to top-performing countries.^26^^,^^27^ Capacity development without local actors and locally relevant data will not guarantee that issues in the Global South with worldwide relevance will be tackled (i.e., the loss of species in tropical biomes). Similarly, other consortia in the Global South like the African BioGenome Project have faced similar challenges, like higher operational costs, difficult logistics for transporting samples, and insufficient capacity to translate genomic research into commercial products.^28^ We strongly encourage that international funding bodies and DSI benefit sharing consider going beyond their geopolitical jurisdiction to enable local scientists to tackle pressing global issues where it is most needed while avoiding the current status that centralizes the generation and analyses of genomic data in more developed countries or regions (Figure 3). Therefore, we encourage global biodiversity funding agencies to support conservation efforts in developing countries also through financing biodiversity genomics under KMGBF’s target 20 (“strengthen capacity-building, technology transfer, and scientific and technical cooperation for biodiversity”).^29^

What is next for the future of genomics in Brazil? We believe that expanding the network of scientists involved, capacity development, production of high-quality data, and collaborations to process data will catalyze a wider interest in genetic data associated with species monitoring, genetic improvement, and applied conservation. GBB’s commitment to take the data produced directly to research groups, organizations, and private companies involved in acting toward conservation, biodiversity monitoring, and bioeconomy development will provide traction for the expansion of the network, as we will be able to demonstrate the value of such an effort. We wish to provide a collection of genomes and data that are of scientific relevance but also actionable.

Brazil is at the leading edge of conservation and is primed for the application and diffusion of the importance of genomics. The Amazon Summit happened in August 2023 in Belém, and the city will also host the 30^th^ United Nations Framework Convention on Climate Change (UNFCCC COP 30) in 2025. Given the global importance of the biodiversity contained in this continental-scale country, we urge a sustained government and private investment in biodiversity genomics as a means of policymaking and implementation of best practices for conservation, ensuring long-term evolutionary resilience and preparation for future climate changes.

## Acknowledgments

The authors are immensely grateful to all the collaborators of the GBB consortium. Funding for the GBB consortium is provided by Vale. A.A. acknowledges support from a Productivity Research Fellowship from the Brazilian Council for Scientific and Technological Development (10.13039/501100003593CNPq, #309243/2023-8).

## Author contributions

Conceptualization, S.T.V. and A.A.; analysis, B.M.S. and H.V.F.; writing – original draft, S.T.V. and A.A.; writing – review & editing, A.F.V., A.C.D.P., C.S.C., C.P., D.L.-L., G.L.N., I.S.M., J.A.P.B., L.G.C., L.E.C.C., R.R.M.O., R.P.D., S.V., S.B.B., V.T., G.O., and A.B.M.; project administration, A.B.M. and A.A.

## Declaration of interests

The authors declare no competing interests.
